# Integrated clinicopathological, genomic, and immunophenotypic landscape of renal tubulocystic oncocytoma

**DOI:** 10.3389/fimmu.2026.1728022

**Published:** 2026-04-14

**Authors:** Wenchi Wang, Hengxing Zhu, Chunyu Chen, Man Sun, Honglong Wang, Yu Tai, Sishan Chen, Zhuo Liu, Bo Fan, Yinghua Li

**Affiliations:** 1 Department of Oncology, Second Affiliated Hospital of Dalian Medical University, Dalian, Liaoning, China; 2 Department of Clinical Medicine, Second Clinical School of Dalian Medical University, Dalian, Liaoning, China; 3 Department of Urology, Second Affiliated Hospital of Dalian Medical University, Dalian, Liaoning, China; 4 Institute of Cancer Stem Cell, Cancer Center, Dalian Medical University, Dalian, Liaoning, China; 5 Department of Emergency Medicine, Shengjing Hospital of China Medical University, Shenyang, Liaoning, China; 6 Department of Pathology, Dalian Friendship Hospital, Dalian, Liaoning, China; 7 Department of Anesthesiology, Dalian Medical University, Dalian, Liaoning, China; 8 Department of Urology, Peking University Third Hospital, Beijing, China

**Keywords:** genomic landscape, immunohistochemistry, renal tubulocystic oncocytoma, tumor microenvironment, whole-exome sequencing

## Abstract

**Background:**

Renal tubulocystic oncocytoma (RTO) is an exceptionally rare variant of renal oncocytoma (RO) with poorly understood genetic underpinnings. This study aimed to characterize the clinicopathological features and genomic landscape of RTO to enhance diagnostic precision and elucidate its molecular profile.

**Methods:**

Whole-exome sequencing (WES) was performed on a pathologically confirmed case of RTO to identify somatic mutations. Bioinformatics filtering identified single-nucleotide variants (SNVs) and insertions/deletions (INDELs), which were screened against databases such as the Cancer Gene Census (CGC) to identify potential driver and predisposing genes. Findings were validated via Sanger sequencing. Immunohistochemistry (IHC) was utilized for diagnostic marker assessment and tumor microenvironment (TME) characterization. Furthermore, a literature-based analysis of reported RTO cases was conducted across multiple databases, including PubMed, Ovid, Google Scholar, EMBASE, and Scopus.

**Results:**

WES analysis identified 399 somatic SNVs and 91 INDELs, as well as mutations in 14 candidate predisposing genes and 27 candidate driver genes, including mutations in 3 predisposing genes (COL7A1, CSF3R, and MKL1) and 11 driver genes (ZFHX3, TSC2, NFATC2, TCF7L2, TLR4, RANBP17, ITK, NEB [Chr2:152499355], NUP214, FBN2, and NEB [Chr2:152544903]). Drug-target prediction and resistance analysis identified several variants with potential therapeutic relevance. IHC staining confirmed positive expression of CD117, EMA, and E-cadherin, supporting the differential diagnosis. TME profiling revealed an “immune-cold” phenotype characterized by low densities of CD4+, CD8+, and FOXP3+ T cells, CD19+ and CD20+ B cells, CD56+ and CD57+ NK cells, and CD163+ tumor-associated macrophages, alongside minimal checkpoint activity and focal fibroblast activation. A comparative analysis with eight previously reported cases further contextualized these clinicopathological and immunophenotypic findings.

**Conclusions:**

This study provides a comprehensive genomic characterization of RTO. The integration of molecular profiling, histopathology, and literature-based comparison broadens the understanding of RTO’s molecular and immunophenotypic landscape, providing a foundation for future hypothesis-driven research.

## 1 Introduction

Renal tubulocystic oncocytoma (RTO) is an extremely rare benign renal neoplasm characterized by a distinctive tubulocystic architecture and oncocytic cell differentiation (1). As a newly recognized histologic subtype of renal oncocytoma (RO), RTO accounts for approximately 3–7% of all RO cases (1–3). Due to its distinct tubulocystic morphology and prominent cystic components, RTO is often radiologically indistinguishable from malignant cystic renal cell carcinomas (RCC), particularly the cystic variant of clear cell RCC or multilocular cystic renal neoplasms of low malignant potential (3, 4). This diagnostic overlap frequently results in preoperative misclassification and unnecessary surgical intervention.

RTO typically presents as a well-circumscribed, multilocular tubulocystic mass without the classic imaging signs of conventional RO, such as central stellate scar or homogeneous enhancement (1, 2). The Bosniak classification system, while effective for stratifying renal cystic lesions, cannot reliably distinguish benign entities like RTO from malignant cystic RCCs, especially in Bosniak category III lesions, which carry a substantial risk of malignancy but may still include benign mimics (5). Despite advances in imaging modalities and biopsy techniques, definitive diagnosis still relies on postoperative histopathology and immunohistochemistry (6, 7).

Histologically, RTO consists of tightly packed eosinophilic cells arranged in a distinctive tubulocystic pattern embedded within edematous or myxoid stroma (1, 8). The tumor demonstrates a benign architectural appearance, lacking features such as nuclear atypia, necrosis, or invasive growth (6). Immunohistochemically, tumor cells typically show positive expression of CD117, EMA, and E-cadherin, while markers such as CK7, CD10, Vimentin, and AMACR are negative or only weakly expressed (3, 9, 10). This immunoprofile supports differentiation from other eosinophilic renal neoplasms, including chromophobe RCC, papillary RCC, and tubulocystic RCC (1, 6).

To date, only a limited number of RTO cases have been reported in the literature, and the genetic landscape of this rare entity remains poorly understood. In this study, we present a case of RTO that was initially misdiagnosed as cystic RCC based on radiological findings. We provide a comprehensive analysis of its clinicopathological and immunohistochemical features, summarize previously reported RTO cases, and, for the first time, characterize its somatic mutational landscape using whole-exome sequencing (WES), identifying potential driver and predisposing genes. These findings may offer novel insights into the pathogenesis and diagnostic distinction of this uncommon tumor type.

## 2 Methods

### 2.1 Clinical study

#### 2.1.1 Immunohistochemical analysis

A renal tumor specimen was obtained from the pathology laboratory of our hospital. The tissue was fixed in 10% neutral buffered formalin, embedded in paraffin, and sectioned into 4 μm slices. Selected sections were stained with hematoxylin and eosin (HE) and examined under a light microscope for histopathological evaluation. Other sections were subjected to immunohistochemical staining with various antibodies. Diagnostic markers included CD117, CD10, E-cadherin, CK7, EMA, Vimentin, AMACR (P504S), and Ki-67, which were used to assess the epithelial nature, renal origin, and proliferative activity of the tumor. To characterize the tumor microenvironment (TME), additional immunostaining was performed for markers of tumor-associated macrophages (CD163 and CD68), regulatory T cells (FOXP3), T lymphocytes (CD4 and CD8), B lymphocytes (CD19 and CD20), natural killer (NK) cells (CD56 and CD57), endothelial cells (CD31 and CD34), fibroblasts (α-smooth muscle actin [SMA]), and immune checkpoint protein PD-L1. All staining procedures followed standard immunohistochemistry protocols. Detailed information on the primary antibodies used is provided in **Supplementary Table 1**. Positive control tissues were used to verify antibody specificity for diagnostic markers, while adjacent normal renal tissue served as a reference for tumor microenvironment analysis. Immunohistochemical staining was semi-quantitatively assessed using the modified H-score method. Fifteen random fields of view were examined at 400× magnification. Staining intensity was graded as 0 (negative), 1 (weak), 2 (moderate), or 3 (strong), and the percentage of positive cells was estimated. The H-score was calculated as follows: H-score = (1 × % weak) + (2 × % moderate) + (3 × % strong). Intergroup comparisons of H-score data were performed using R (version 4.2.1), utilizing the ggplot2 (v3.4.4), stats (v4.2.1), and car (v3.1-0) packages. Statistical significance was determined using Student’s t-test.

#### 2.1.2 Literature review of reported RTO cases

To contextualize our findings, a literature review was conducted. We searched PubMed, Ovid, EMBASE, Scopus, and Google Scholar up to August 2025 using the terms “renal tubulocystic oncocytoma” and the combination of “renal oncocytoma” AND “tubulocystic”. Cases were included if sufficient clinical, radiological, histopathological, and immunohistochemical data were reported. Eight previously published cases met these criteria. Data extraction included patient demographics, tumor size and location, treatment, clinical outcomes, pathology, and immunoprofile. The eight reported cases, together with our case, were summarized in **Table 1**; **Supplementary Tables S2**, **S3**.

**Table 1:** Patient’s characteristics of reported cases of RTO.

Author	Case no.	Sex	Age (years)	Clinical manifestation	Tumor site	Tumor size(cm)	Primary diagnosis	Treatment	Follow-up (months)	Outcome
Zhang Q, 2015	1	Female	42	Physical examination	Right upper pole	3.5	RCC	Laparoscope, Partial nephrectomy	10	No evidence of disease
Zhao M, 2016	2	Male	74	Dysuria	Left	3	RCC	Laparoscope, Partial nephrectomy	6	No evidence of disease
Xiong B, 2022	3	Female	74	Hypertension, Dizziness	Right middle pole	1.5	RCC	Laparoscope, Partial nephrectomy	NA	NA
Al-Delfi F, 2016	4	Female	64	Incidental	Right	2.9	Right renal mass	Robotic-assisted, Partial nephrectomy	NA	NA
He HY, 2018	5	Female	59	NA	NA	1.5	NA	NA	76	No evidence of disease
	6	Male	25	NA	NA	3.8	NA	NA	12	No evidence of disease
X Leroy, 2006	7	Male	67	Microscopic haematuria	Left	7	NA	Radical nephrectomy	26	No evidence of disease
	8	Female	47	Abdominal pain	Left upper pole	3.5	NA	Partial nephrectomy	8	No evidence of disease
Our case	9	Female	59	Chest tightness, Abdominal pain	Left upper pole	2	Cystic RCC	Laparoscope, Partial nephrectomy	36	No evidence of disease

NA, not available; RCC, renal cell carcinoma.

### 2.2 Whole-exome sequencing

#### 2.2.1 DNA extraction

According to the manufacturer’s instructions isolation of sample genomic DNA was performed using the GeneRead DNA Extraction Kit (Qiagen, Germany). DNA degradation and RNA contamination were analyzed by agarose gel electrophoresis, DNA purity was measured by Nanodrop (OD260/280 ratio), and DNA concentration was accurately quantified by Qubit^®^ 2.0 Fluorometer (Invitrogen, USA).

#### 2.2.2 Library preparation and sequencing

Genomic DNA was randomly sheared into 180–280 bp fragments using a Covaris system (Covaris, Massachusetts, USA). Whole-exome capture and library construction were performed using the Agilent SureSelect Human All Exon kit (Agilent Technologies, CA, USA), strictly following the manufacturer’s instructions and optimized protocols. Captured DNA libraries were amplified by PCR, quality-checked, and sequenced on the Illumina HiSeq platform (Illumina, CA, USA) by Novogene (Beijing, China).

#### 2.2.3 Quality control

Raw sequencing data were subjected to stringent quality control to ensure the reliability of downstream analysis. Reads containing adapter sequences, those with more than 10% unidentified bases (N), and those with low-quality bases (Phred score <5) accounting for over 50% of the read length were removed. Quality filtering was performed using standard pipelines, and high-quality clean reads were retained. The overall quality metrics met standard thresholds, including Q30 values above 80% and average base error rates below 1%, with most reads showing a GC content within the expected range. These clean reads were used in all subsequent analyses.

#### 2.2.4 WES data processing and mutation analysis

SNPs and INDELs were identified using SAMtools (v1.0) based on the aligned BAM files. SNPs were categorized by mutation type (e.g., synonymous, missense, stopgain, stoploss) and genomic location (e.g., CDS, UTRs, intronic, intergenic), and the transition/transversion ratio was calculated to assess variant quality. INDELs were also classified according to their functional consequences, including frameshift and non-frameshift insertions/deletions. Somatic SNVs and somatic INDELs were called using MuTect and Strelka, respectively. High-confidence somatic mutations were retained based on standard filtering criteria (e.g., depth, base quality, and variant allele frequency).

All identified variants were functionally annotated using ANNOVAR. The annotation process included gene-based classification, prediction of amino acid changes, known variant database cross-referencing (e.g., dbSNP, ClinVar), and pathogenicity scoring (e.g., SIFT, PolyPhen-2, CADD). Variants were further interpreted in the context of potential cancer relevance.

#### 2.2.5 Bioinformatics analysis

Clean reads were aligned to the human reference genome (GRCh37/hg19) using BWA (11), and BAM files were generated and processed with Sambamba (v0.4.7) to mark duplicate reads. SNP and INDEL variants were detected using SAMtools (v1.0) (12), and functional annotation was performed with ANNOVAR (13). Cancer-related predisposing and known driver genes were identified based on CGC and other public databases. Circos was used to visualize the mutation landscape.

#### 2.2.6 Analysis of potential driver mutations and predisposing genes

Variants detected in the available normal tissue were compared with the CGC database to screen for potential cancer predisposing genes. Somatic variants identified in the tumor tissue were subsequently compared with known driver gene datasets, including CGC513, Bert Vogelstein125, SMG127, and Comprehensive435, to identify potential driver gene alterations in this case.

#### 2.2.7 Functional enrichment analysis

The identified driver and predisposing genes were subjected to functional enrichment analysis. Gene Ontology (GO) enrichment analysis, covering biological process (BP), cellular component (CC), and molecular function (MF), and Kyoto Encyclopedia of Genes and Genomes (KEGG) pathway analysis were performed using the “clusterProfiler” R package (version 4.3.1). A q value < 0.05 was considered statistically significant.

### 2.3 Sanger sequencing

Sanger sequencing was performed to validate somatic variants identified by WES. Genomic DNA was extracted using a commercial column-based kit (Sangon Biotech, China), and target regions were amplified by PCR with primers designed using Primer Premier 5. PCR products were verified by agarose gel electrophoresis, purified, and sequenced using the Applied Biosystems™ 3730xl DNA Analyzer. Sequencing chromatograms were analyzed using Chromas or SeqMan software.

### 2.4 Drug-gene interaction and resistance prediction

To explore the therapeutic relevance of the somatic mutations identified by WES, we performed drug-target prediction using the NovoDrug database. This platform integrates multiple curated sources, including DrugBank, PharmGKB, PG_FDA, MyCancerGenome, and KEGG, to annotate gene variants with existing targeted drugs. We compared high-frequency somatic mutations in our tumor sample with the NovoDrug reference database, and selected those with matched drug-target associations supported by at least one data source.

To assess potential drug resistance, we used the resistance screening module of NovoDR (the drug resistance database in the NovoDrug platform). Variants were filtered based on quality control thresholds, including a sequencing depth ≥8×. Only exonic and non-synonymous mutations were included for resistance matching. The filtered mutations were aligned with a curated database containing clinically relevant resistance variants across multiple cancer types and drug classes. For each gene, the drug resistance association, cancer context, variant type, and supporting literature were recorded.

## 3 Results

### 3.1 Clinical presentation and imaging evaluation of our case

A 59-year-old female presented with a one-month history of paroxysmal retrosternal chest tightness of unclear etiology; she sought medical attention after experiencing one day of worsening chest pain. The patient had occasional abdominal pain and bloating. She had a 10-year history of hypertension, managed with regular oral nifedipine sustained-release tablets, with blood pressure maintained at 125-140/80–95 mmHg. Electrocardiography (ECG) revealed pathological q waves in leads II, III and AVF. As a result, the patient was initially diagnosed with grade 3 hypertension and arrhythmia (occasional ventricular premature beats).

**Figure 1:**
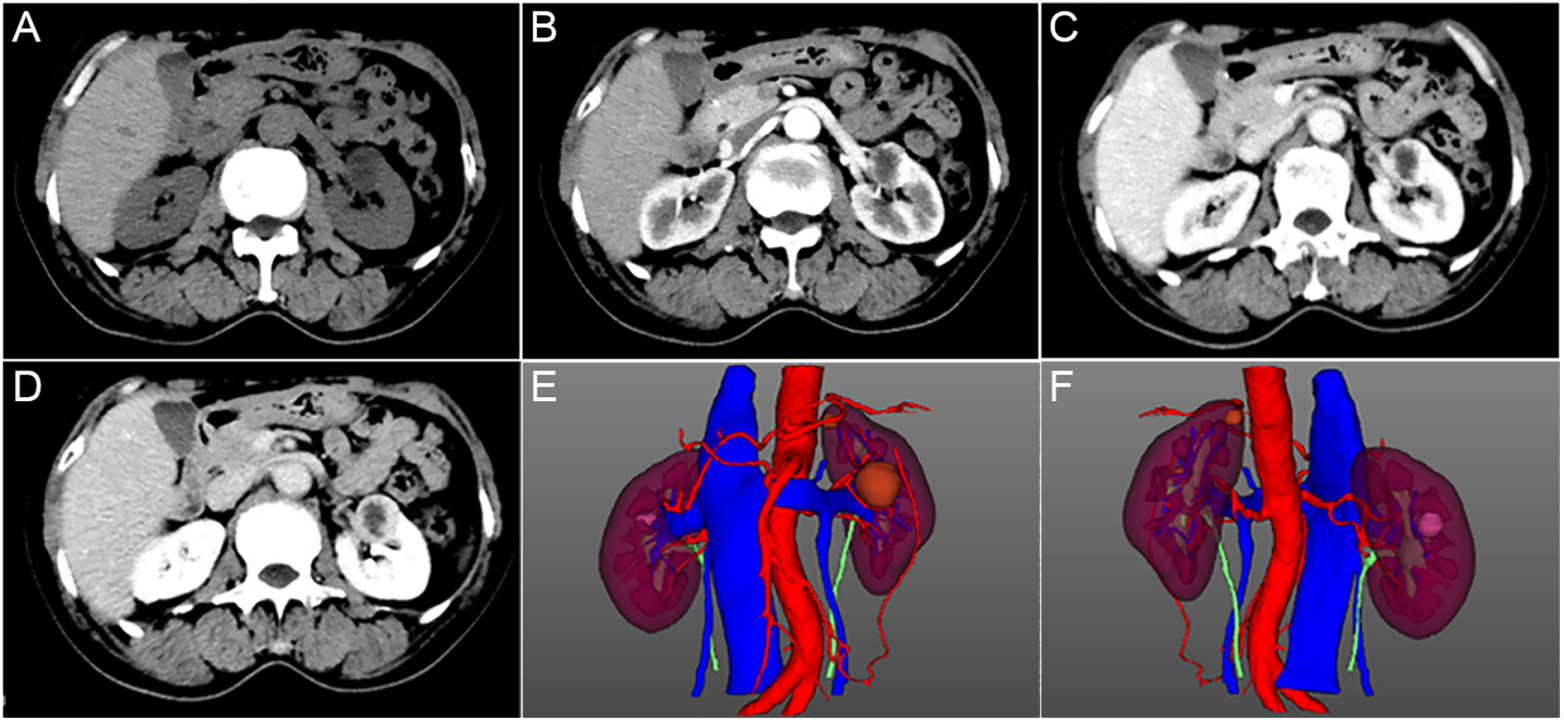
CT imaging and three-dimensional reconstruction. **(A–D)** Enhanced CT of the lower abdomen showing a round-shaped, slightly low-density cyst measuring approximately 24×28 mm in size in the left kidney. The lesion shows peripheral rim enhancement on contrast-enhanced scans. **(E, F)** Three-dimensional images of anterior view and dorsal view are reconstructed.

To evaluate for potential secondary causes of hypertension, imaging investigations were performed. The CT plain scan and CT 3D reconstruction of the adrenal gland identified a thickened left adrenal junction and an approximately 24 × 23 mm round, low-density shadow (12 HU) indicating a renal cyst in the left kidney. Computed tomography angiography (CTA) and coronary reconstruction revealed that the calcium score was zero and there was no abnormity. Subsequent Contrast-enhanced CT confirmed the presence of a well-defined cystic mass (24 × 28 mm) with thin septa and marginal enhancement, suggestive of a multilocular or tubulocystic architecture (**Figures 1A–D**). Moreover, the Contrast-enhanced CT identified a 12 × 10 mm non-enhancing lesion in the right kidney and an 8 mm lesion in the left kidney. Three-dimensional reconstruction of the cystic mass was performed (**Figure 1E, F**). Nodules and an isodense shadow with mild contrast enhancement and small lymph nodes were identified in the retroperitoneal and abdominal cavity areas.

**Figure 2:**
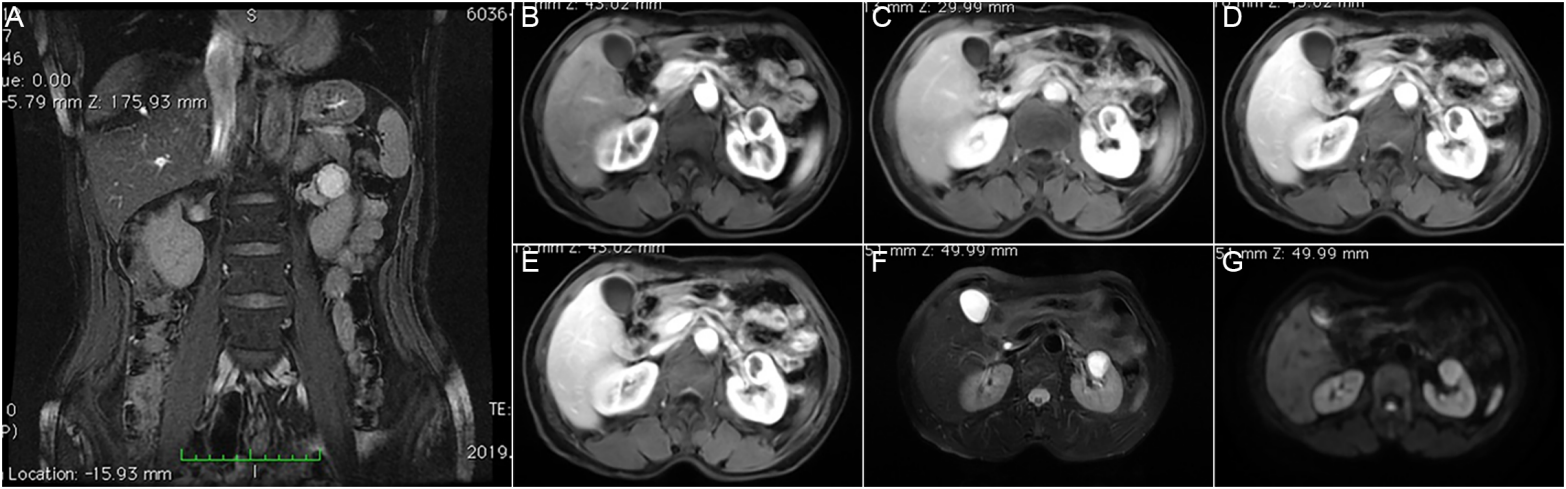
MRI characteristics of the cystic renal lesion. The lesion measured 21 × 18 mm, showing low signal intensity on T1WI, high signal intensity on T2WI and DWI, and peripheral enhancement with internal irregular non-enhanced areas on contrast-enhanced imaging. **(A)** Coronal T2-weighted image. **(B–E)** Axial contrast-enhanced images obtained at different phases. **(F)** Axial T2-weighted image. **(G)** Axial diffusion-weighted image.

Subsequent contrast-enhanced MRI (**Figure 2**) of the left kidney revealed a cystic lesion (21 × 18 mm) with irregular internal septations and peripheral rim enhancement, consistent with a multilocular tubulocystic morphology, with low signal intensity on T1 weighted imaging (T1WI), high signal intensity on T2 weighted imaging (T2WI), and high signal intensity on diffusion weighted imaging (DWI). The bilateral renal parenchyma had an 8-mm rounded area with low signal intensity on T1WI, high signal intensity on T2WI and no enhancement. In addition, color Doppler imaging indicated normal flow in the renal artery. An ultrasonogram of the abdomen revealed a 1.5 × 1.2 cm mass (echoless, clear margin and strong sound transmission in the middle-upper part) in the right kidney and a 2.4 × 2.2 cm mass with a clear margin, mixed echogenicity, thick cyst wall, poor sound transmission due to the presence of fluid, a small number of separated light bands and blood flow around the circumference of the left kidney.

### 3.2 Clinicopathologic and immunohistochemical findings

#### 3.2.1 Histopathological presentation

Cystic RCC of the left kidney was initially suspected based on radiological findings, and the patient underwent laparoscopic partial nephrectomy. Intraoperatively, a 2 × 2 cm exophytic mass was identified at the anterosuperior pole of the left kidney, appearing inconsistent with the typical features of cystic RCC. Gross examination revealed a well-circumscribed lesion with focal hemorrhage and prominent tubulocystic degeneration. The cut surface was grayish-red to grayish-yellow. Histologically, the tumor consisted of oncocytic cells with abundant eosinophilic cytoplasm and prominent nucleoli, arranged in solid, tubular, and tubulocystic patterns. The background stroma was mildly edematous, and no features of nuclear pleomorphism, necrosis, or vascular invasion were observed.

#### 3.2.2 Immunohistochemical profile for diagnosis

**Figure 3:**
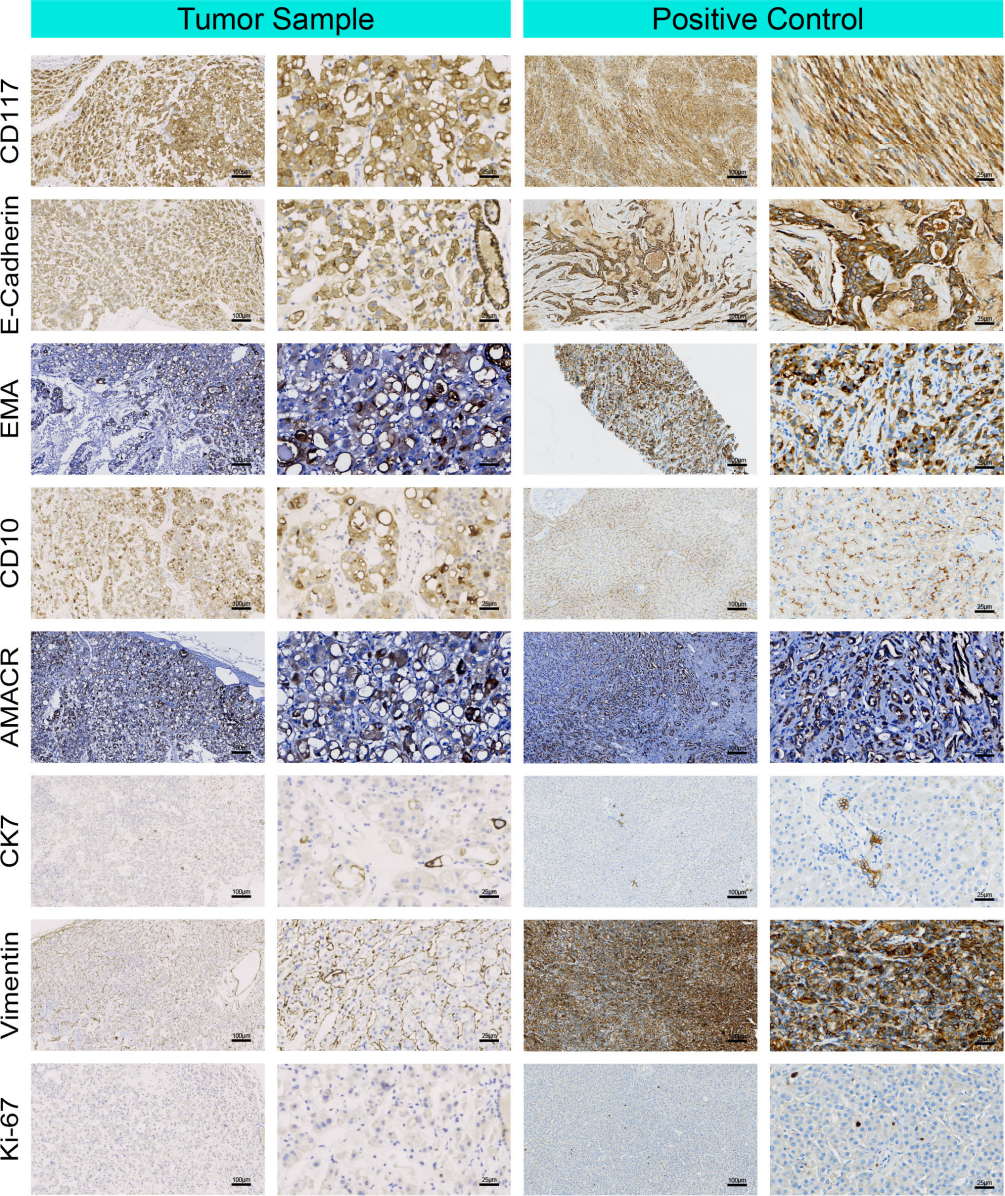
Immunohistochemical profile of RTO. Representative staining of CD117, E-cadherin, EMA, CD10, AMACR, CK7, Vimentin, and Ki-67 in tumor samples compared with positive controls. Positive control tissues were included to verify antibody specificity and the expected staining patterns of the diagnostic markers.

Immunohistochemical staining demonstrated that the tumor cells were diffusely positive for CD117, E-cadherin, and epithelial membrane antigen (EMA), with a Ki-67 proliferation index of ≤1%, indicating low proliferative activity. CD10 and α-methylacyl-CoA racemase (AMACR) exhibited focal positivity. In contrast, the tumor was negative for CK7 and vimentin (**Figure 3**). These markers also assist in the differential diagnosis of eosinophilic renal tumors, as diffuse CK7 expression is characteristic of chromophobe RCC, while CD10, AMACR, and vimentin are commonly expressed in papillary RCC or clear cell RCC. Although focal AMACR expression sometimes overlaps with papillary RCC, the absence of CK7 and vimentin helps exclude papillary RCC and clear cell RCC, respectively (3, 6). In addition, the absence of characteristic chromophobe RCC markers (such as diffuse CK7 positivity) and the co-expression of CD117, EMA, and E-cadherin, together with the distinctive morphology, further support classification as RTO rather than malignant RCC.

#### 3.2.3 Literature review of reported RTO cases

A total of eight previously reported RTO cases were identified through the literature review (1, 8, 9, 14–16). Patient ages ranged from 25 to 74 years, with a slight female predominance (5/8). Tumor sizes varied between 1.5 and 7.0 cm, and most lesions were located in the upper pole of the kidney. Partial nephrectomy was the most common surgical approach. In cases with available follow-up data, no recurrence or metastasis was observed (**Table 1**).

Histologically, all cases displayed the characteristic tubulocystic growth pattern with eosinophilic oncocytic cytoplasm and low-grade nuclei. Thickened cyst walls were frequently described, whereas the central stellate scar typical of conventional RO was largely absent. Additional features such as intraluminal hemorrhage, fibrotic or edematous stroma, and heterogeneous vascularity were variably reported, while mitoses and necrosis were generally absent. Immunohistochemically, RTO cases consistently expressed CD117, EMA, and E-cadherin, with low Ki-67 indices (≤1%). In contrast, CK7 and vimentin were usually negative, while AMACR and CD10 showed variable or focal positivity in a minority of cases. These findings highlight that, although RTO shares an overlapping immunophenotype with RO, its distinctive tubulocystic architecture and occasional atypical marker expression underscore its recognition as a separate entity. Our case (the ninth) demonstrated similar features, further supporting this classification.

#### 3.2.4 Immunohistochemistry for detecting components of the TME

**Figure 4:**
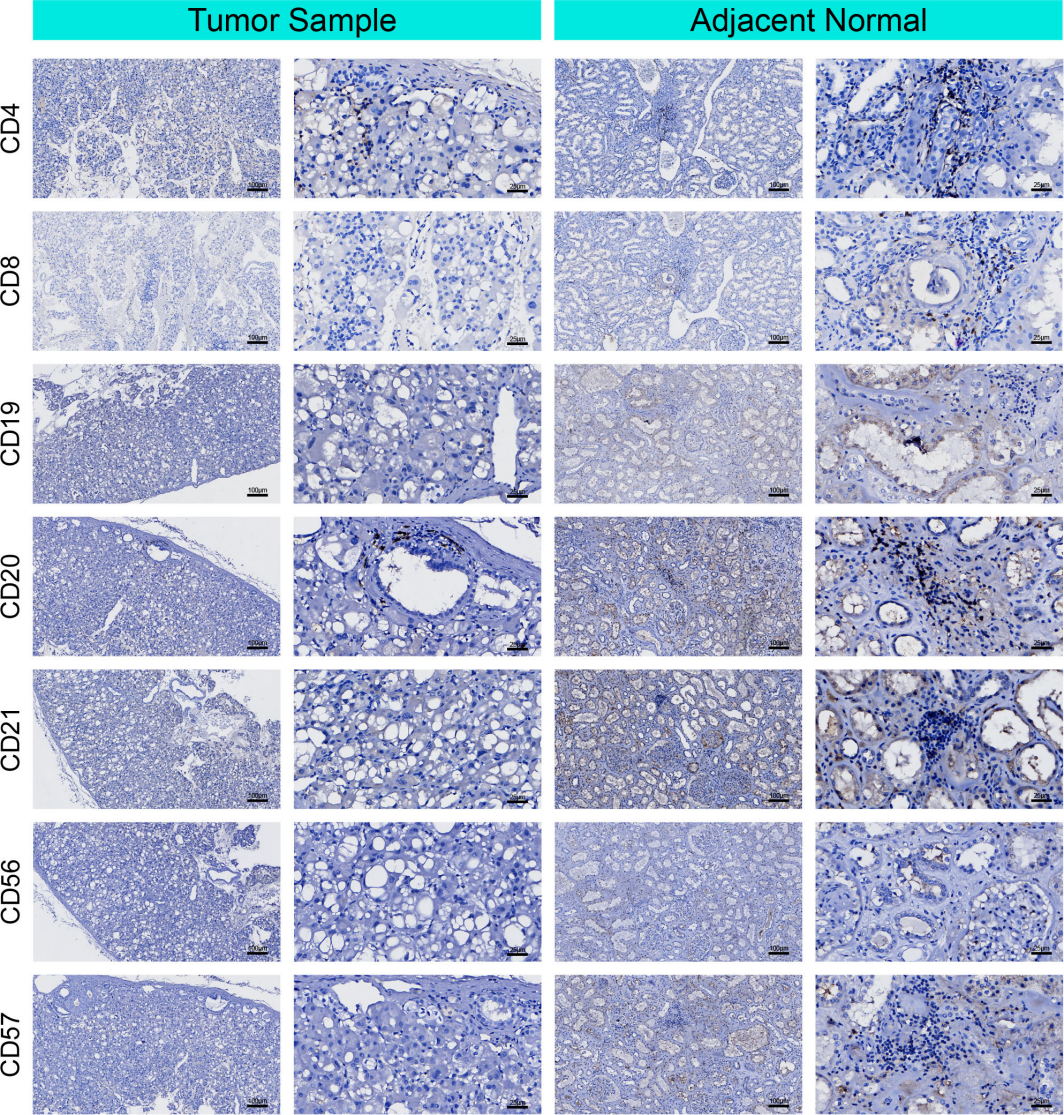
Immunohistochemical profile of immune cell infiltration markers in the tumor microenvironment of RTO. Representative staining of CD4, CD8, CD19, CD20, CD21, CD56, and CD57 in tumor tissue and adjacent normal tissue. Adjacent normal renal tissue served as a reference for comparison of immune cell infiltration patterns.

**Figure 5:**
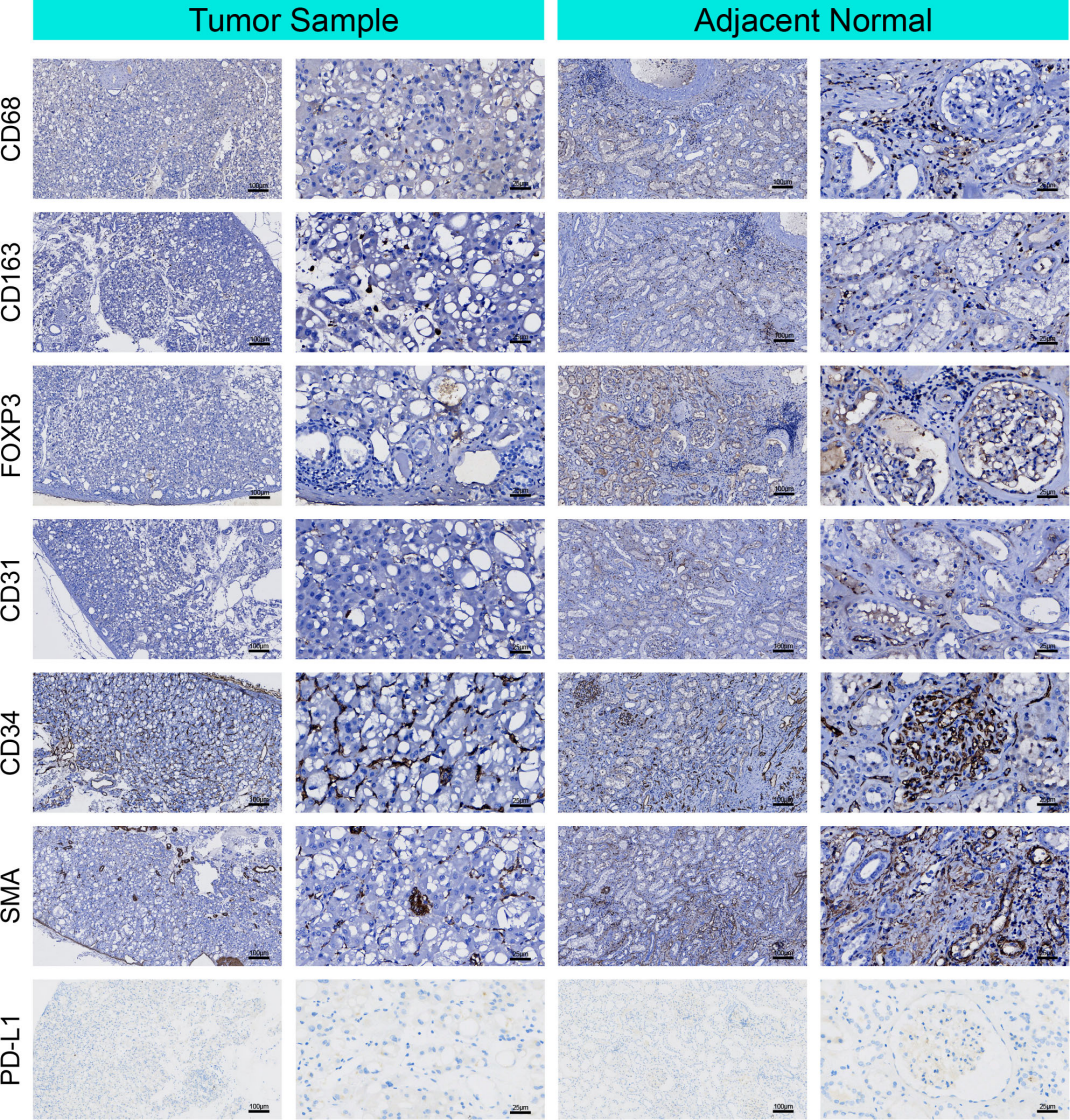
Immunohistochemical staining of immunoregulatory, vascular, and stromal markers in the tumor microenvironment of RTO. Representative staining of CD68, CD163, FOXP3, CD31, CD34, SMA, and PD-L1 in tumor tissue and adjacent normal tissue. Adjacent normal renal tissue served as a reference for comparison of immune, vascular and stromal features.

To better characterize the TME of RTO, which may provide insights into tumor biology and potential immune regulatory features, immunohistochemical staining was performed to evaluate immune, vascular, and stromal components. Compared with adjacent normal tissue, RTO demonstrated reduced infiltration of CD4+ and CD8+ T cells, CD19+/CD20+/CD21+ B-cell–related markers, and CD56+/CD57+ NK cells (**Figure 4**). FOXP3+ regulatory T cells and CD163+ macrophages were present at relatively low density, predominantly localized to the peritumoral stroma, whereas CD68+ macrophages showed relatively higher expression in tumor tissue than in adjacent normal tissue. Endothelial markers CD31 and CD34 exhibited weak and patchy staining, consistent with a low microvessel density. SMA highlighted focal activation of stromal fibroblasts surrounding cystic and tubular areas. PD-L1 expression was weak and focal, observed in scattered tumor cells and stromal elements without diffuse distribution (**Figure 5**). Overall, these findings depict a TME with low immune cell infiltration, limited vascularization, and focal stromal activity.

Semi-quantitative analysis using the modified H-score method further supported these observations. Quantitative comparison demonstrated that the H-scores of immune markers CD4, CD8, CD19, CD20, CD21, CD56, and CD57 were significantly lower in tumor tissue than in adjacent normal tissue (P < 0.001). Similarly, macrophage- and stromal-related markers including CD163, FOXP3, CD31, and SMA also showed significant differences between tumor and adjacent tissues (P < 0.001). The distribution patterns of H-scores are illustrated in **Supplementary Figures S2**, **S3**.

### 3.3 Whole-exome sequencing analysis

#### 3.3.1 WES identification of SNPs and INDELs

To explore the potential pathogenic mechanisms of RTO, WES was performed on tumor samples. A total of 88,191 SNPs were identified, with most located in intronic and coding (CDS) regions. Among the exonic SNPs, synonymous and missense mutations were the most prevalent (**Supplementary Figure 1A**). Transition (Ts) and transversion (Tv) mutations accounted for 62,153 and 26,038 of the total SNPs, respectively, yielding a Ts/Tv ratio of 2.39. Of these, 760 variants were novel (not recorded in dbSNP), with a novel Ts/Tv ratio of 1.52 (**Supplementary Table 4**). In addition, there are about 350K INDELs in a whole human genome, and 11995 INDELs were found in the RTO sample, concentrated in intron regions. Coding region INDELs were mainly nonframeshift deletions and insertions (**Supplementary Figure 1B**). Among all INDELs, 1,738 were novel (not annotated in dbSNP), accounting for approximately 14.5% of the total. The overall dbSNP annotation rate was 85.51%, suggesting a relatively high proportion of known variants in this sample (**Supplementary Table 5**).

#### 3.3.2 Analysis of somatic SNVs and INDELs

Somatic mutations, non-inherited mutations arising in somatic cells, play a key role in tumorigenesis (17). Therefore, we investigated somatic mutations in RTO samples. A total of 399 somatic SNVs were identified using MuTect, all located in the coding sequence (CDS) region. The majority were missense mutations (n = 387), followed by 12 stopgain mutations (**Supplementary Table 6**). Additionally, 91 somatic INDELs were detected by Strelka, all located within CDS regions. The most common types were nonframeshift deletions (n = 35) and nonframeshift insertions (n = 36) (**Supplementary Table 7**).

#### 3.3.3 Analysis of predisposing and driver genes

**Figure 6:**
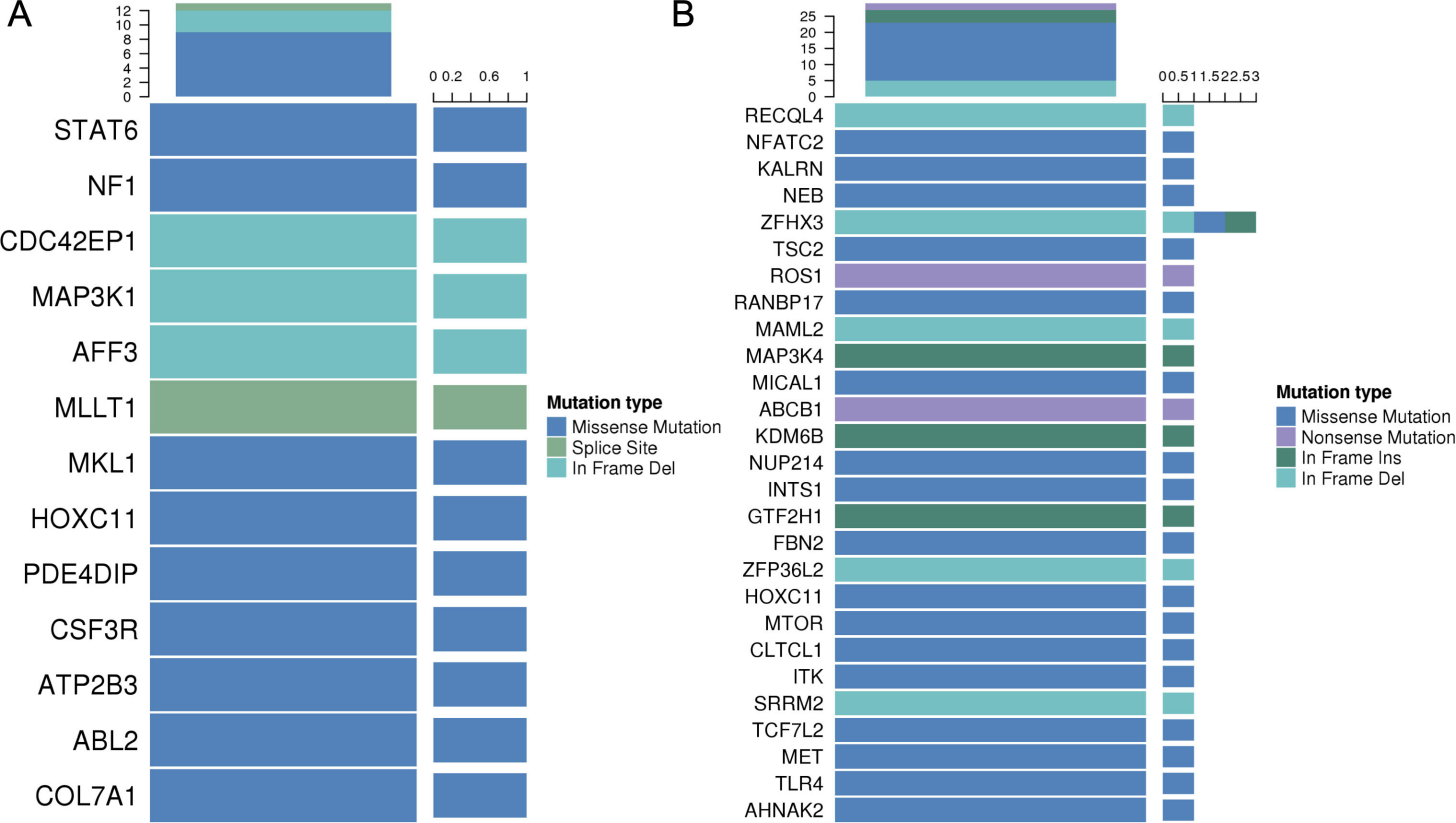
Genetic alterations in RTO. **(A)** Candidate predisposing genes. **(B)** Known driver gene analysis.

Cancer predisposing genes can confer inherited or acquired susceptibility to tumorigenesis under certain environmental or genetic contexts. By comparing somatic mutations in the RTO sample with the CGC database, we identified 14 potential cancer predisposing genes, including ABL2, NF1, MLLT1, COL7A1, STAT6, CSF3R, ATP2B3, PDE4DIP (Chr1:144916748), PDE4DIP (Chr1:144922583), MKL1, HOXC11, CDC42EP1, AFF3, and MAP3K1 (**Figure 6A**). We compared the somatic mutations with known driver genes to identify driver gene alterations in the RTO sample (**Figure 6B**). Using four driver gene resources (CGC, BertVogelstein125, SMG127, and Comprehensive435), we compiled a list of candidate driver genes. Among them, seven genes (MICAL1, AHNAK2, ZFHX3, CLTCL1, TSC2, ABCB1, and NFATC2) were selected for further analysis based on their annotation as high-confidence drivers in at least one authoritative source and their plausible biological relevance (**Table 2**).

**Table 2:** The analysis results of the known driver genes.

Hugo_ symbol	Chromosome	Position	Ref_allele	Alt_ allele	Variant_ classification	Amino acid change	CGC	BertVogelstein125	Comprehensive435	SMG127
MICAL1	6	109769459	A	C	Missense_Mutation	MICAL1:NM_001159291:exon12:c.T1544G:p.I515S|MICAL1:NM_001286613:exon13:c.T1859G:p.I620S|MICAL1:NM_022765:exon13:c.T1802G:p.I601S	-	-	Candidate driver	-
AHNAK2	14	105407525	G	A	Missense_Mutation	AHNAK2:NM_138420:exon7:c.C14263T:p.P4755S	-	-	High Confidence Driver	-
ZFHX3	16	72828843	T	A	Missense_Mutation	ZFHX3:NM_001164766:exon8:c.A4996T:p.S1666C|ZFHX3:NM_006885:exon9:c.A7738T:p.S2580C	-	-	High Confidence Driver	-
CLTCL1	22	19211534	G	C	Missense_Mutation	CLTCL1:NM_001835:exon14:c.C2172G:p.D724E|CLTCL1:NM_007098:exon14:c.C2172G:p.D724E	ALCL	-	High Confidence Driver	-
TSC2	16	2106720	A	G	Missense_Mutation	TSC2:NM_000548:exon8:c.A724G:p.T242A|TSC2:NM_001077183:exon8:c.A724G:p.T242A|TSC2:NM_001114382:exon8:c.A724G:p.T242A	“Pulmonary lymphangioleiomyomatosis (LAM), renal angiomyolipoma, HNSCC” “Hamartoma, renal cell carcinoma, tuberous sclerosis tuber”	-	High Confidence Driver	-
ABCB1	7	87160730	C	T	Nonsense_Mutation	ABCB1:NM_000927:exon22:c.G2565A:p.W855X	-	-	High Confidence Driver	-
NFATC2	20	50092193	T	C	Missense_Mutation	NFATC2:NM_001136021:exon4:c.A1277G:p.H426R|NFATC2:NM_001258292:exon4:c.A1277G:p.H426R|NFATC2:NM_001258294:exon4:c.A680G:p.H227R|NFATC2:NM_001258295:exon4:c.A680G:p.H227R|NFATC2:NM_001258296:exon4:c.A680G:p.H227R|NFATC2:NM_001258297:exon4:c.A680G:p.H227R|NFATC2:NM_012340:exon4:c.A1337G:p.H446R|NFATC2:NM_173091:exon4:c.A1337G:p.H446R	Ewing sarcoma	-	-	-

#### 3.3.4 Functional enrichment analysis

**Figure 7:**
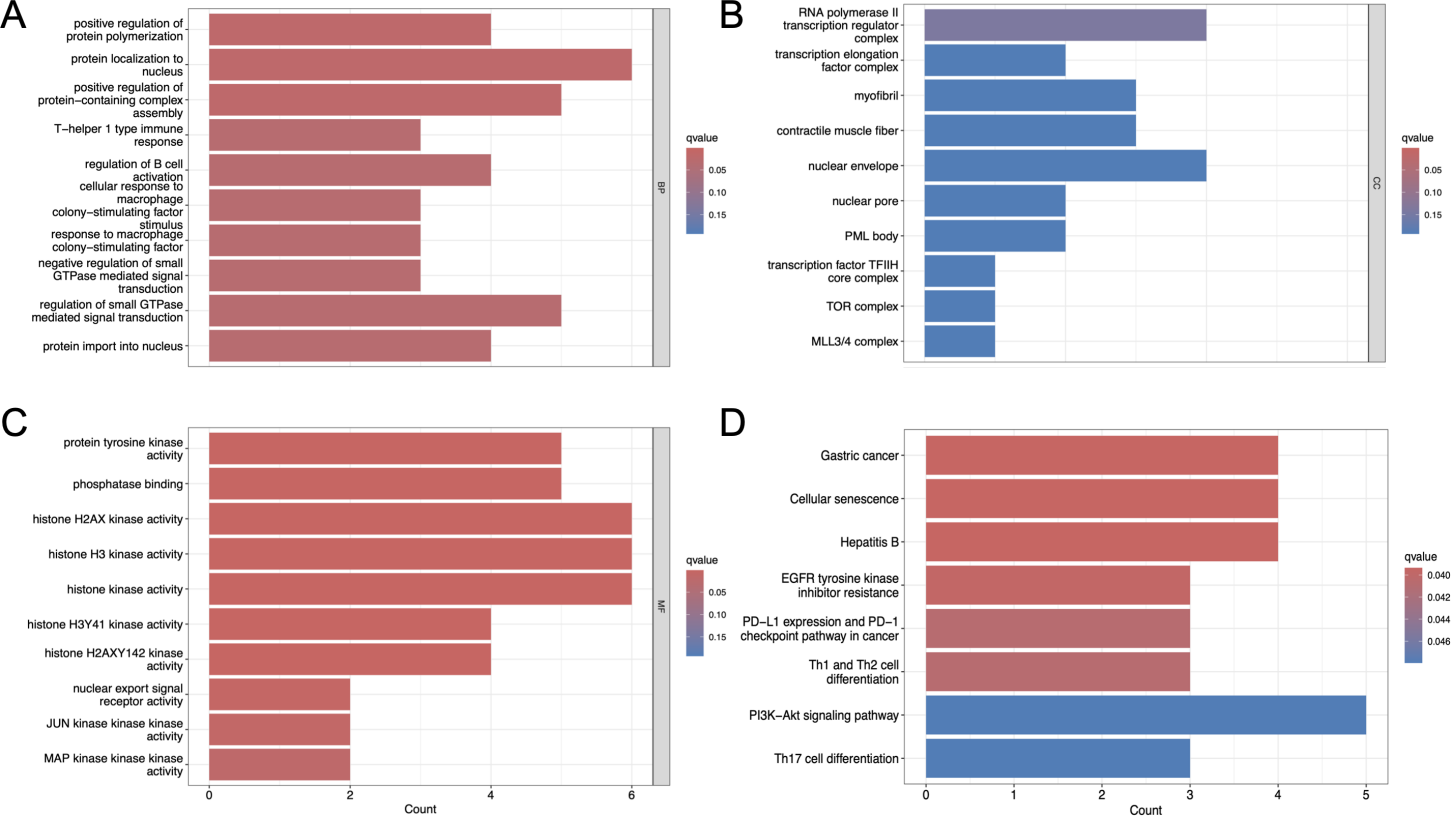
Functional enrichment of mutated genes in RTO. **(A)** GO biological process terms. **(B)** GO cellular component terms. **(C)** GO molecular function terms. **(D)** KEGG pathway enrichment analysis.

Functional enrichment of the candidate predisposing and driver genes showed close links to immune-related GO categories, such as regulation of B-cell activation, T-helper cell differentiation, and responses to macrophage colony-stimulating factor (**Figures 7A–C**). At the cellular component and molecular function levels, associations were also observed with chromatin- and transcription factor- related complexes, as well as DNA-binding and kinase activity. KEGG pathway mapping additionally pointed to T-cell receptor signaling, Th1/Th2/Th17 differentiation, and the PD-L1/PD-1 checkpoint axis (**Figure 7D**). Other relevant pathways included PI3K-Akt signaling, mechanisms of EGFR inhibitor resistance, and chromatin remodeling. Collectively, these results suggest that the mutated genes converge on processes involving immune regulation, canonical oncogenic cascades, and transcriptional control.

#### 3.3.5 Sanger sequencing

**Figure 8:**
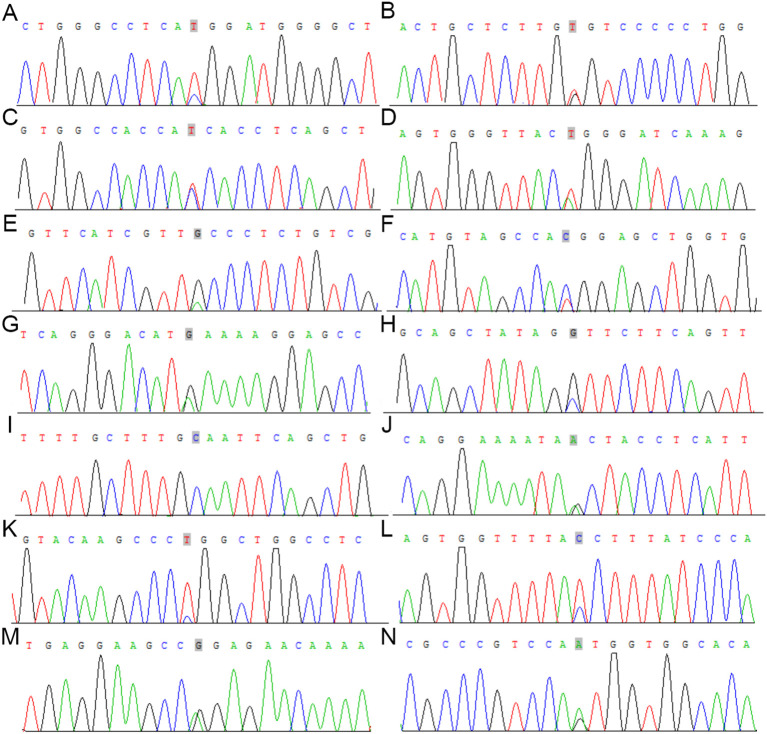
Validation of mutations by Sanger sequencing in RTO. **(A-N)** Variants are indicated by a gray box. **(A)** COL7A1 **(B)** CSF3R **(C)** MKL1 **(D)** ZFHX3 **(E)** TSC2 **(F)** NFATC2 **(G)** TCF7L2 **(H)** TLR4 **(I)** NEB **(J)** RANBP17 **(K)** ITK **(L)** NEB **(M)** NUP214 **(N)** FBN2.

**Figure 9:**
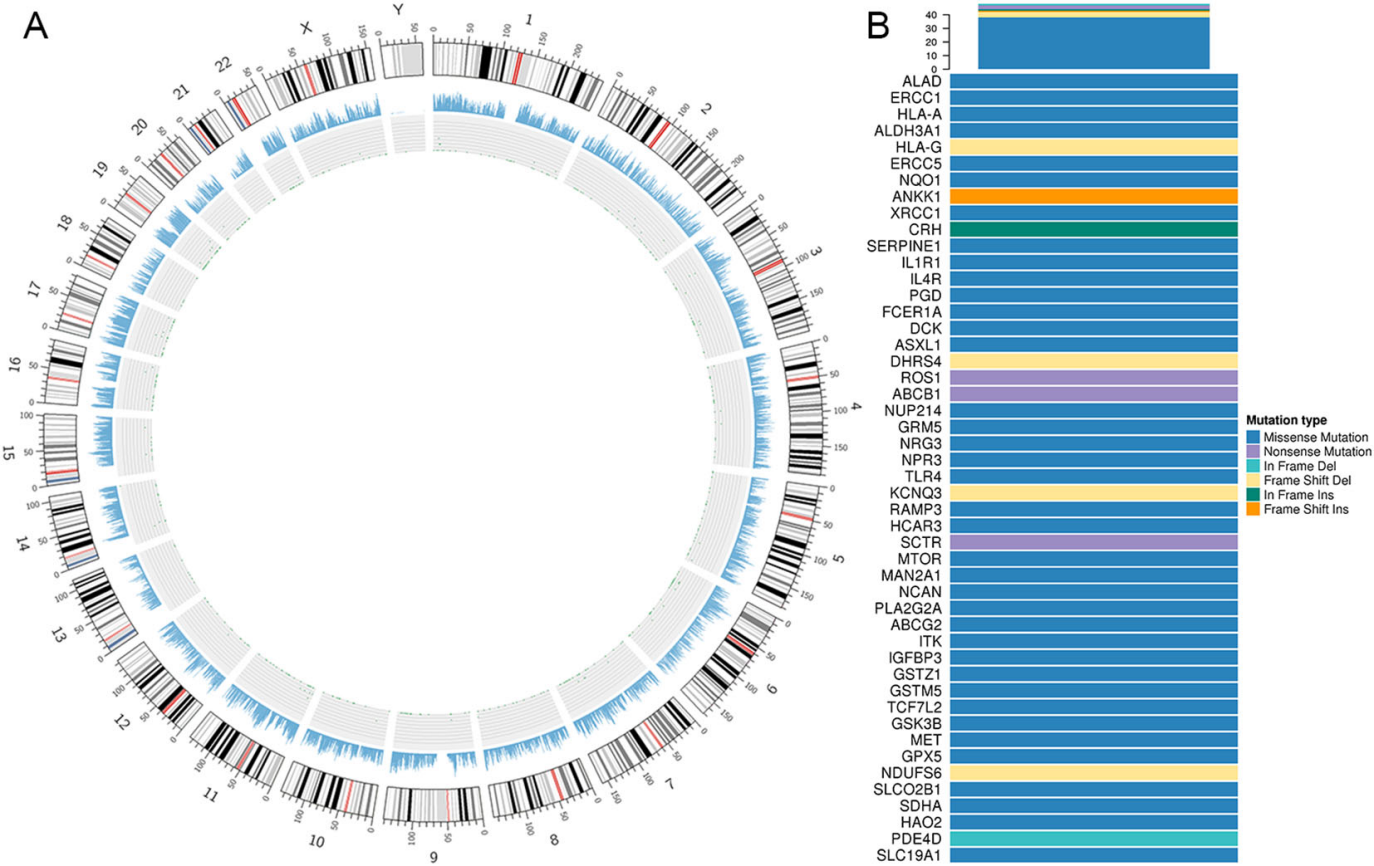
Genomic landscape of RTO. **(A)** Circos plot showing sequencing coverage and distribution of SNPs/INDELs. **(B)** High-frequency mutated genes mutated in RTO samples in the NovoDrug database.

To confirm the variants discovered by WES, Sanger sequencing was applied. A total of 14 mutations were confirmed, including 13 heterozygous variants and 1 homozygous variant. Heterozygous mutations were detected in the following genes: COL7A1 at Chr3: 48623782 T>C, CSF3R at Chr1: 36932272 T>G, MKL1 at Chr22: 40815148 T>C, ZFHX3 at Chr16: 72828843 T>A, TSC2 at Chr16: 2106720 A>G, NFATC2 at Chr20: 50092193 T>C, TCF7L2 at Chr10: 114849251 A>G, TLR4 at Chr9: 120470966 C>G, RANBP17 at Chr5: 170336704 A>G, ITK at Chr5: 156675967 T>C, NEB at Chr2: 152499355 T>C, NUP214 at Chr9: 134027192 A>G, and FBN2 at Chr5: 127800515 A>G. A single homozygous variant was also observed in NEB (Chr2:152544903 C>C) (**Figure 8**). A Circos diagram was then produced to display the overall distribution and local density of somatic mutations across the genome, showing scattered but distinct clusters of mutational enrichment (**Figure 9A**).

#### 3.3.6 Analysis of targeted drug predictions and resistance mutations

Targeted therapies offer more precise intervention at specific mutations in tumor cells, reducing toxicity and enhancing efficacy. By comparing high-frequency mutated genes in RTO samples with the NovoDrug database, we identified several genes with potential targeted drug associations, including ABCB1, FCER1A, ROS1, NQO1, and MAN2A1. Predicted agents include ondansetron, omalizumab, anti-EGFR antibodies, dicumarol, and swainsonine (an experimental inhibitor) (**Figure 9B**; **Supplementary Table 8**). The NovoDrug platform integrates multiple pharmacogenomic resources, such as PharmGKB, My Cancer Genome, and KEGG, and includes evidence from clinical trials (ClinicalTrials.gov), thereby providing a comprehensive annotation of drug-mutation relationships.

To further investigate resistance-related alterations, we analyzed somatic mutations using the NovoDR drug resistance mutation database. Five genes (ERCC1, MET, HIF1A, ABCB1, and ABCG2) were identified to be associated with resistance to a range of anticancer drugs. For example, ERCC1 was associated with cisplatin resistance, while MET and HIF1A were linked to resistance to bevacizumab or gefitinib. ABCG2 and ABCB1 mutations were associated with resistance to 19 and 27 drugs, respectively (**Supplementary Table 9**). These results outline the potential for personalized therapy in RTO patients by highlighting candidate druggable mutations and known resistance variants.

## 4 Discussion

RTO represents a rare benign renal neoplasm with a characteristic combination of oncocytic morphology and tubulocystic growth. In our patient, preoperative imaging revealed a multiloculated cystic renal mass containing hemorrhagic fluid, closely mimicking malignant cystic RCC, particularly cystic clear cell RCC. MRI demonstrated heterogeneous signal intensity with enhancing mural nodules and diffusion restriction, features that strongly suggested malignancy and complicated preoperative diagnosis. Although renal biopsy could theoretically improve diagnostic accuracy in similarly challenging cystic renal lesions (18), procedural risks, including hemorrhage, pseudoaneurysm formation, tumor seeding, and inadequate sampling due to limited viable cellular components in cystic tumors (19), often outweigh its benefits. Recently, imaging innovations such as superb microvascular imaging (SMI) and dual-energy CT (DECT) have been explored as noninvasive options (20–22). SMI significantly enhances visualization of blood flow within cystic septations and solid mural components (20, 22), while DECT effectively reduces pseudo-enhancement artifacts, facilitating differentiation between hemorrhagic or proteinaceous cysts and neoplastic lesions (21). Nevertheless, despite these technological improvements, differentiating benign cystic tumors like RTO from malignant RCC in Bosniak category III-IV lesions remains difficult, underscoring the need for definitive histopathological confirmation after resection.

A synthesis of our case with previously reported RTO cases highlights several key clinicopathological insights. RTO spans a broad age range, frequently presenting in middle-aged and elderly individuals without a clear sex predilection, and typically manifests as a small, well-circumscribed lesion with multilocular tubulocystic architecture. These features, combined with nonspecific clinical presentations and the absence of the classical central stellate scar seen in conventional oncocytoma, explain why RTO is often misclassified as cystic RCC preoperatively. Histologically, the tumor’s characteristic tubulocystic growth pattern, thickened cyst walls, and oncocytic cytoplasm with low-grade nuclei contrast sharply with malignant RCC, which typically shows necrosis, vascular invasion, and high mitotic activity. Immunohistochemistry further refines this diagnostic distinction: across published cases and in our own, RTO consistently expresses CD117, EMA, and E-cadherin with a uniformly low Ki-67 index, while showing recurrent negativity for CK7 and vimentin and only variable CD10 or AMACR positivity. Although focal CD10 or AMACR staining may occasionally lead to diagnostic ambiguity, the co-expression of definitive oncocytic markers, in combination with distinctive tubulocystic morphology, supports the classification of RTO as a benign oncocytic neoplasm rather than a malignant RCC variant. Importantly, evaluation of the tumor microenvironment revealed only sparse immune infiltration and limited vascularization, consistent with the indolent clinical course of RTO and providing further contrast with the angiogenic and immunologically active microenvironment typically seen in RCC.

Additionally, another tumor entity recognized by the World Health Organization, tubulocystic renal cell carcinoma (TCRCC), may exhibit partially overlapping histologic features with RTO, particularly in cases of cystic architecture (3, 23, 24). Both tumors share a tubulocystic growth pattern and oncocytic cytoplasm, which may pose a diagnostic challenge, especially in limited biopsy samples. However, key morphologic differences aid in their distinction. TCRCC typically exhibits higher nucleolar grades, more compact fibrotic stroma, and frequent presence of mitotic figures and microscopic necrosis, which are rarely observed in RTO (3). Importantly, RTO often contains solid nests or islands of tumor cells embedded within loose or myxoid stroma, features largely absent in TCRCC (25). Immunohistochemically, CD117 is diffusely positive in RTO but generally negative in TCRCC. Conversely, TCRCC tends to show stronger expression of vimentin, CD10, AMACR, and CK7, alongside a significantly higher Ki-67 proliferation index (26). Awareness of these morphologic and immunophenotypic distinctions is crucial to avoid misdiagnosis and ensure appropriate patient management.

Several candidate driver genes identified in this study, including ZFHX3, TSC2, NFATC2, TCF7L2, TLR4, and ITK, are known to participate in transcriptional regulation, immune signaling, and mTOR-related pathways. For example, TSC2 plays a key role in regulating mTOR signaling, while NFATC2, TLR4, and ITK are involved in immune activation and inflammatory signaling (27–34). In addition, RANBP17 and NUP214, which regulate nucleocytoplasmic transport, and FBN2, an extracellular matrix component, may influence cellular signaling and stromal interactions (35–38). Furthermore, mutations in cancer predisposing genes such as COL7A1, MKL1, and CSF3R, which are involved in extracellular matrix integrity, cytoskeletal regulation, and hematopoietic signaling (38–43). Although these genes are not classically associated with renal tumors, they may suggest potential stromal–immune interactions in RTO.

Taken together, these driver and predisposing gene alterations suggests that pathways related to mTOR signaling, transcriptional regulation, nucleocytoplasmic transport, and extracellular matrix remodeling may collectively contribute to the biological characteristics of RTO. Although the exact mechanisms underlying the characteristic tubulocystic morphology of RTO remain unclear, several hypotheses can be proposed. The consistent oncocytic phenotype suggests a potential origin from distal nephron or collecting duct epithelium, in which aberrant differentiation may promote tubular and cystic expansion (44). Accumulation of mitochondria, a hallmark of oncocytomas, may contribute to altered energy metabolism and epithelial polarity, favoring cystic growth (45, 46). Moreover, stromal reactions such as fibrosis and edema likely support the maintenance of thickened cyst walls (47). These findings collectively suggest that RTO formation may result from the interplay of epithelial metabolic reprogramming, stromal remodeling, and altered signaling pathways.

At the pathway level, functional enrichment analysis further indicated that the identified genes were enriched in several immune-related pathways, including Th1/Th2/Th17 differentiation and the PD-L1/PD-1 checkpoint axis. This is consistent with our IHC observation of limited PD-L1 expression and sparse immune infiltration. In addition, enrichment in PI3K-Akt and EGFR-related pathways suggests partial overlap with canonical oncogenic signaling observed in RCC, despite the benign clinical behavior of RTO (48). Drug-target prediction analysis also suggested that several mutations may be associated with potential drug-related pathways; however, given the indolent nature of RTO, these observations should be interpreted cautiously and primarily as exploratory molecular insights rather than immediate therapeutic implications.

While this study offers comprehensive clinical, pathological, and genetic insights into RTO, it is inherently limited by its single-case design. Larger, multicenter cohorts are essential to more fully characterize the clinical behavior and genomic landscape of this entity. Future research integrating single-cell sequencing, spatial transcriptomics, and advanced imaging-based deep learning may provide pivotal insights, enhancing diagnostic precision and facilitating less invasive management strategies.

## 5 Conclusion

In this study, we conducted a comprehensive analysis of an extremely rare case of RTO characterized by tightly packed eosinophilic cells in a distinctive tubulocystic pattern. Immunohistochemical staining showed positivity for CD117, E-cadherin, and EMA, with focal positivity for CD10 and AMACR and negativity for CK7, aiding diagnosis and differential diagnosis. WES identified several novel mutations, including candidate predisposing genes (COL7A1, CSF3R, MKL1) and potential driver genes (e.g., TSC2, NFATC2, TCF7L2, TLR4, and ITK), with 14 variants confirmed by Sanger sequencing. We also reviewed previous RTO cases to summarize shared clinical, pathological, and immunohistochemical features. These findings broaden current understanding of the clinicopathological and molecular characteristics of RTO and provide preliminary insights into its molecular features.

## Data Availability

The datasets presented in this study can be found in online repositories. The names of the repository/repositories and accession number(s) can be found below: https://figshare.com/10.6084/m9.figshare.30392998.
