# Supplementary material for: Integrated clinicopathological, genomic, and immunophenotypic landscape of renal tubulocystic oncocytoma
Source: Front Immunol. 2026 Apr 14;17:1728022. doi: 10.3389/fimmu.2026.1728022 (PMC13120908; doi:10.3389/fimmu.2026.1728022)
Supplement: Supplementary file 1 [file DataSheet1.docx]

Supplementary Material

**Table S1**. Antibodies used in the immunohistochemistry. Ig, Immunoglobulin; CD, cluster of differentiation.

| **Antibody** | **Species** | **Antibody number** | **Source** | **Dilution** |
| --- | --- | --- | --- | --- |
| c-Kit  (CD117) | Rabbit monoclonal | 34-8800 | ThermoFisher Scientific, Massachusetts, USA | 1:50 |
| E-cadherin | Rabbit monoclonal | ab40772 | Abcam, Cambridge, UK | 1:500 |
| EMA | Mouse monoclonal | MA5- 11202 | ThermoFisher Scientific, Massachusetts, USA | 1:200 |
| Vimentin | Rabbit monoclonal | ab92547 | Abcam, Cambridge, UK | 1: 400 |
| CD10 | Mouse monoclonal | MA5-15231 | ThermoFisher Scientific, Massachusetts, USA | 1: 400 |
| Cytokeratin 7 (CK7) | Mouse monoclonal | ab9021 | Abcam, Cambridge, UK | 1:400 |
| AMACR | Rabbit monoclonal | MA5-44595 | ThermoFisher Scientific, Massachusetts, USA | 1:1000 |
| Ki-67 | Rabbit polyclonal | 27309-1-AP | Protein Tech Group, Chicago, USA | 1:6000 |
| CD4 | Rabbit monoclonal | ab183685 | Abcam, Cambridge, UK | 1:500 |
| CD8 | Mouse monoclonal | 66868-1-Ig | Protein Tech Group, Chicago, USA | 1:6000 |
| CD19 | Rabbit monoclonal | ab245235 | Abcam, Cambridge, UK | 1:1000 |
| CD20 | Rabbit monoclonal | ab78237 | Abcam, Cambridge, UK | 1:100 |
| CD56 | Mouse monoclonal | MA1-06801 | ThermoFisher Scientific, Massachusetts, USA | 1:200 |
| CD57 | Mouse monoclonal | 27087-MSM4-P1 | ThermoFisher Scientific, Massachusetts, USA | 2 μg ml^−1^ |
| CD163 | Rabbit monoclonal | ab182422 | Abcam, Cambridge, UK | 1: 400 |
| CD68 | Mouse monoclonal | ab955 | Abcam, Cambridge, UK | 1: 3000 |
| FOXP3 | Rabbit monoclonal | 14-5773-82 | ThermoFisher Scientific, Massachusetts, USA | 1:200 |
| PD-L1 | Rabbit monoclonal | ab205921 | Abcam, Cambridge, UK | 2 μg ml^−1^ |
| CD31 | Rabbit polyclonal | ab28364 | Abcam, Cambridge, UK | 1:50 |
| CD34 | Rabbit monoclonal | ab110643 | Abcam, Cambridge, UK | 1:50 |

**Table S2.** Pathological features of reported cases of RTO.

| Author | Case NO. | Central Scar | Properties of Nuclear | | | Properties of Mass | | | Properties of Cystic | | |
| --- | --- | --- | --- | --- | --- | --- | --- | --- | --- | --- | --- |
|  |  |  | Nuclear Shape | Nucleolus | Mitosis | Mass Heterogeneity | Solid Component | Vascular Condition | Cyst Wall | Septa | Cyst Content |
| Zhang Q, 2015 | 1 | No | Round, low-grade | One or several | Absent | Yes | Yes | NA | Thicken | Multilocular | Hemorrhagic |
| Zhao M, 2016 | 2 | No | Round, low-grade | Small | Absent | Yes | Yes | No | Thicken | Multilocular | Hemorrhagic |
| Xiong B, 2022 | 3 | No | Round | NA | NA | Yes | Yes | NA | NA | NA | Hemorrhagic |
| Al-Delfi F, 2016 | 4 | Yes | Round | Unconspicuous | NA | Yes | yes | NA | NA | Multilocular | NA |
| He HY, 2018 | 5 | NA | Round | Conspicuous and enlarged | Absent | Yes | No | Hypervascular | Thicken | Multilocular | Hemorrhagic |
|  | 6 | NA | Round | Conspicuous and enlarged | Absent | Yes | No | Hypervascular | Thicken | Multilocular | Hemorrhagic |
| X Leroy, 2006 | 7 | No | Round | One or several | Rare | Yes | Yes | No | Thicken | Multilocular | NA |
|  | 8 | No | Round | One or several | Rare | Yes | Yes | NA | Thicken | Multilocular | NA |
| Our case | 9 | No | Round | One or several | Rare | Yes | Yes | Hypervascular | Thicken | Multilocular | Hemorrhagic |

NA, not available;**Table S3.** Immunohistochemical features of reported cases of RTO.

| Author | Case NO. | IHC marker | | | | | | | |
| --- | --- | --- | --- | --- | --- | --- | --- | --- | --- |
|  |  | CD117 | CK7 | E-cadherin | EMA | Vimentin | AMACR | CD10 | ki-67 |
| Zhang Q, 2015 | 1 | + | - | + | + | - | - | - | 1% |
| Zhao M, 2016 | 2 | + | - | + | + | - | + | - | NA |
| Xiong B, 2022 | 3 | + | - | NA | NA | - | NA | - | 1% |
| Al-Delfi F, 2016 | 4 | NA | + | NA | NA | - | - | - | NA |
| He HY, 2018 | 5 | + | - | NA | NA | - | + | + | ≤1% |
|  | 6 | + | - | NA | NA | - | + | + | ≤1% |
| X Leroy, 2006 | 7 | NA | + | NA | + | NA | NA | - | NA |
|  | 8 | NA | + | NA | + | NA | NA | - | NA |
| Our case | 9 | + | - | + | + | - | + | + | ≤1% |

IHC, immunohistochemical; CK7, cytokeratin 7; AMACR, Alpha-methylacyl-CoA racemase; EMA, epithelial membrane antigen; NA, not available;

**Table S4.** Characterization of the SNPs on the genome.

| **Sample** | **Cro_Tumor** |
| --- | --- |
| Total | 88191 |
| Het | 50050 |
| Hom | 38141 |
| Transition | 62153 |
| Transvertion | 26038 |
| Ts/Tv | 2.39 |
| dbsnp Percentage | 87431 (99.14%) |
| Novel | 760 |
| Novel Ts | 459 |
| Novel Tv | 301 |
| Novel Ts/Tv | 1.52 |

**Table S5.** Characterization of the INDELs on the genome.

| **Sample** | **Cro_Tumor** |
| --- | --- |
| Total | 11995 |
| Het | 6243 |
| Hom | 5752 |
| dbsnp Percentage | 10257 (85.51%) |
| Novel | 1738 |

**Table S6.** Number of Somatic SNVs on different regions of the genome.

| **Sample** | **Cro_Tumor** |
| --- | --- |
| CDS | 399 |
| Synonymous_SNP | 0 |
| Missense_SNP | 387 |
| Stopgain | 12 |
| Stoploss | 0 |
| Unknown | 0 |
| Intronic | 0 |
| UTR3 | 0 |
| UTR5 | 0 |
| Splicing | 0 |
| ncRNA_Exonic | 0 |
| ncRNA_Intronic | 0 |
| ncRNA_UTR3 | 0 |
| ncRNA_UTR5 | 0 |
| ncRNA_Splicing | 0 |
| Upstream | 0 |
| Downstream | 0 |
| Intergenic | 0 |
| Others | 0 |
| Total | 399 |

**Table S7.** Results of InDel detection of somatic cells on different regions of the genome

| **Sample** | **Cro_Tumor** |
| --- | --- |
| CDS | 91 |
| frameshift_deletion | 10 |
| frameshift_insertion | 8 |
| nonframeshift_deletion | 35 |
| nonframeshift_insertion | 36 |
| stopgain | 2 |
| stoploss | 0 |
| unknown | 0 |
| intronic | 0 |
| UTR3 | 0 |
| UTR5 | 0 |
| splicing | 0 |
| ncRNA_exonic | 0 |
| ncRNA_intronic | 0 |
| ncRNA_UTR3 | 0 |
| ncRNA_UTR5 | 0 |
| ncRNA_splicing | 0 |
| upstream | 0 |
| downstream | 0 |
| intergenic | 0 |
| Others | 0 |
| Total | 91 |

**Table S8.** Targeted drug prediction for high-frequency mutation genes

| **Gene** | **Drug** | **PG_FDA** | **DrugBank** | **PharmGKB** | **MyCancerGenome** | **Sources** |
| --- | --- | --- | --- | --- | --- | --- |
| FCER1A | Benzylpenicilloyl polylysine | - | Y | - | - | 1 |
| ROS1 | Anti-EGFR antibodies | - | - | - | Y | 1 |
| MAN2A1 | Swainsonine | - | Y | - | - | 1 |
| NQO1 | Dicumarol | - | Y | - | - | 1 |
| ABCB1 | Ondansetron | - | - | Y | - | 1 |
| ABCB1 | HMG CoA reductase inhibitors | - | - | Y | - | 1 |
| FCER1A | Omalizumab | - | Y | - | - | 1 |

Y, Yes; -, No.

**Table S9.** Results of mutation screening of drug resistance genes.

| **Gene_Symbol** | **Entrez_Gene_Id** | **Chromosome** | **Start_ position** | **End_ Position** | **Num_Drug** | **Drug_Resistance** | **Num_ Cancer** | **Cancer** | **Sample** | **Variant_Classification** | **Reference** |
| --- | --- | --- | --- | --- | --- | --- | --- | --- | --- | --- | --- |
| ERCC1 | 2067 | 19 | 45924602 | 45924602 | 1 | Cisplatin | 3 | Small cell lung cancer, Gastric cancer , Ovarian cancer | Cro_Tumor | Missense_Mutation | PUBMED:21440950 |
| MET | 4233 | 7 | 116339673 | 116339673 | 2 | Bevacizumab, Gefitinib | 2 | Glioblastoma, Non small cell lung cancer | Cro_Tumor | Missense_Mutation | PUBMED:23307858;PUBMED:17463250;PUBMED:25065853;PUBMED:18836087 |
| HIF1A | 3091 | 14 | 62207557 | 62207557 | 1 | Bevacizumab | 1 | Glioblastomas | Cro_Tumor | Missense_Mutation | PUBMED:10188075;PUBMED:22447568 |
| ABCG2 | 9429 | 4 | 89061029 | 89061029 | 19 | Taxanes, Topoisomerase inhhibitors,Imatinib, Erlotinib, Sunitinib, Nilotinib,Mitoxantrone,Doxorubicin, Daunorubicin, Topotecan, SN-38,Epirubicin, Etoposide, Tenipodise, Imatinib, Bisantrene, Gefitinib, Methotrexate, | 7 | Breast cancer, Leukaemia,  Acute myelocytic leukemia,  Edometrial cancer, Esophageal cancer, Colon cancer, Melanoma | Cro_Tumor | Missense_Mutation | PUBMED:11902585;PUBMED:9850061;PUBMED:2564696 |
| ABCB1 | 5243 | 7 | 87160730 | 87160730 | 27 | Taxanes, Topoisomerase inhhibitors,Imatinib, Erlotinib, Sunitinib, Nilotinib,Paclitaxel, Doxorubicin, Vinblastine,Daunorubicin, Acrionmycin-D, Docetaxel, Etoposide, Teniposide, Bisantrene, Homoharringtonine, Bisantrene, Mitoxantrone, Vincristine, Idaunorubicin, Epirubicin, Imatinib, Mitomycin C, Docetaxel, Irinotecan, Topotecan, | 6 | Kidney cancer,  Colon cancer,  Liver cancer,  Prostate cancer, Breast cancer. Leukaemia, | Cro_Tumor | Nonsense_Mutation | PUBMED:10331089;PUBMED:2564696 |


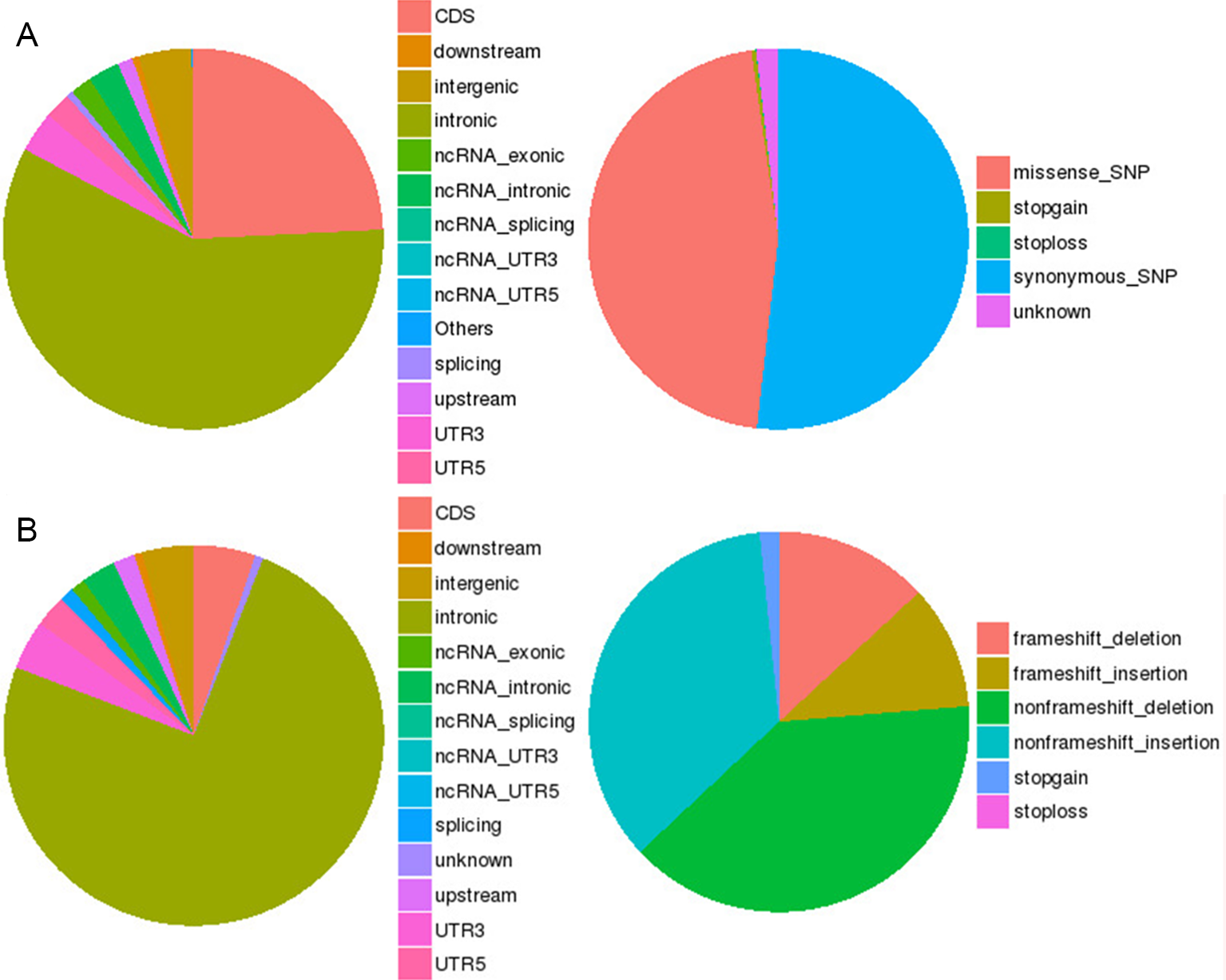


**Figure S1.** (A) Distribution of the number of SNPs in different regions of the genome (left) and the number of different types of SNPs in the coding region (right). (B)Distribution of the number of INDELs in different regions of the genome (left) and the number of different types of INDELs in the coding region (right).


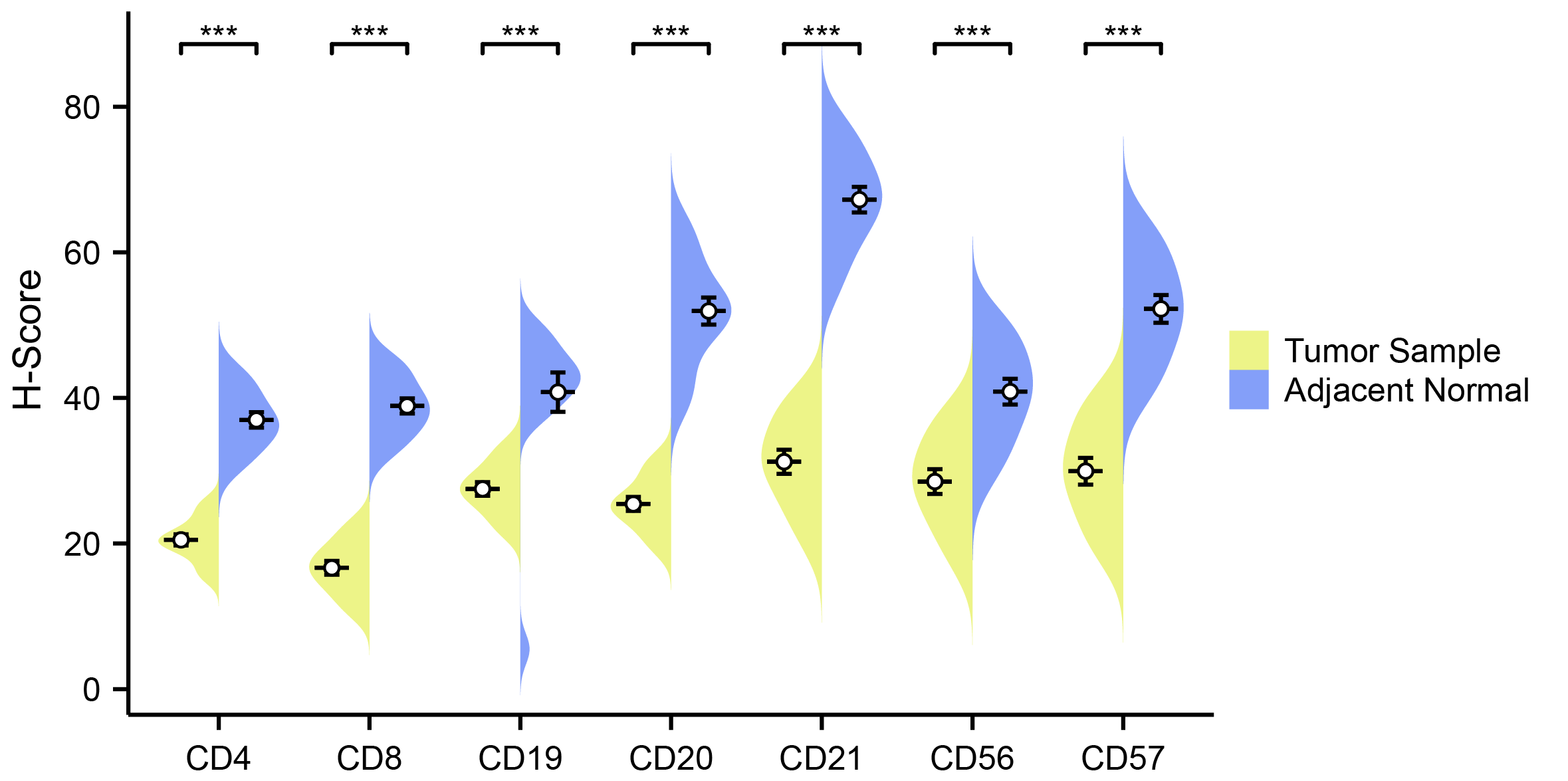


**Figure S2.** Semi-quantitative H-score analysis of immune cell markers in the tumor microenvironment. Distribution of H-scores for CD4, CD8, CD19, CD20, CD21, CD56, and CD57 in tumor tissue and adjacent normal renal tissue.


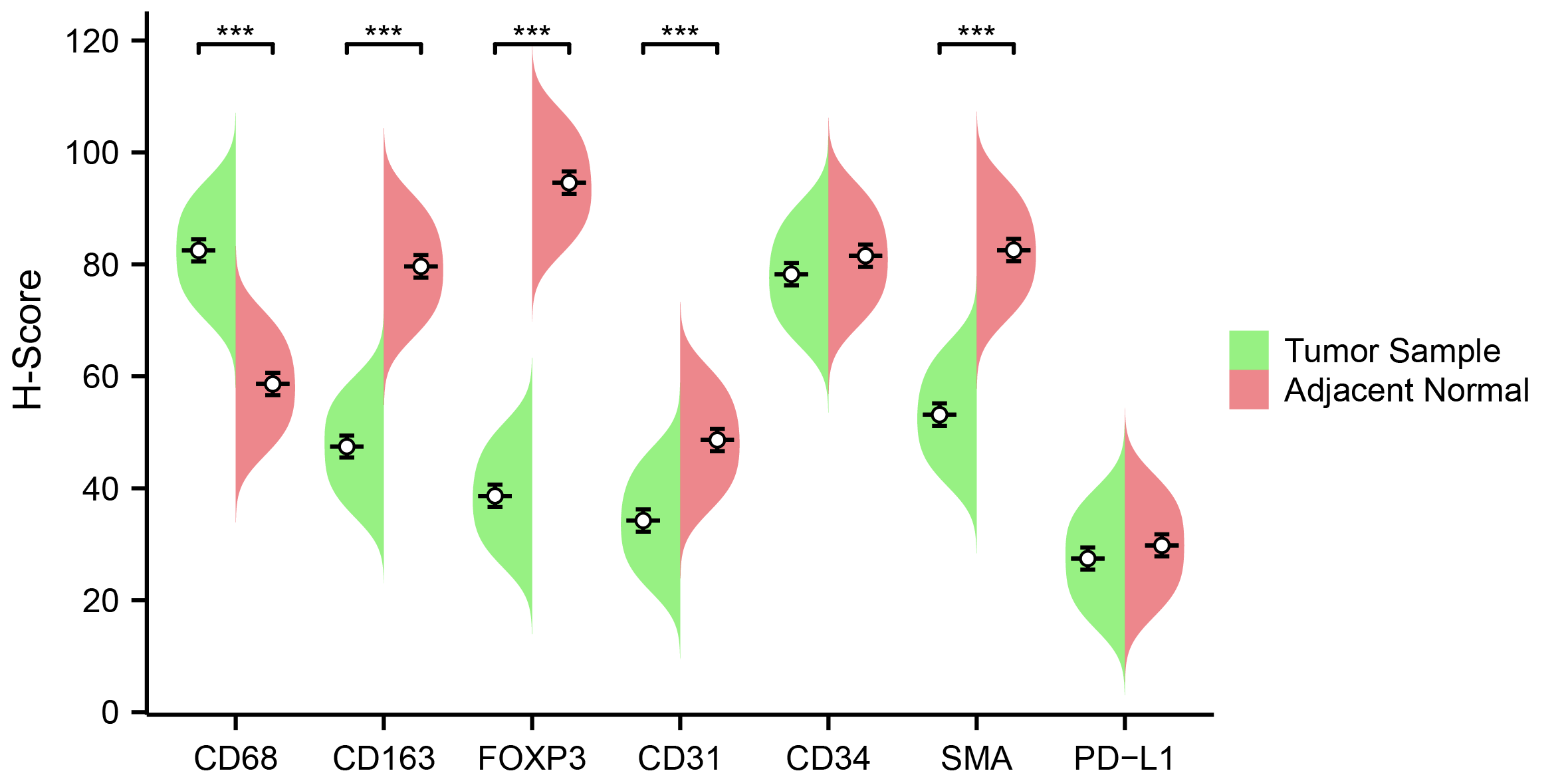


**Figure S3.** Semi-quantitative H-score analysis of immunoregulatory, vascular, and stromal markers. Distribution of H-scores for CD68, CD163, FOXP3, CD31, CD34, SMA, and PD-L1 in tumor tissue and adjacent normal renal tissue.
